# Di-μ_2_-bromido-bis­[bromido(η^6^-1,2,4,5-tetra­methyl­benzene)ruthenium(II)]

**DOI:** 10.1107/S1600536809049642

**Published:** 2009-11-28

**Authors:** Noel Espinosa-Jalapa, Simón Hernández-Ortega, Ronan Le Lagadec, David Morales-Morales

**Affiliations:** a Instituto de Química, Universidad Nacional Autónoma de México, Circuito Exterior, Ciudad Universitaria, México 04510, Mexico

## Abstract

The asymmetric unit of the title compound, [Ru_2_Br_4_(C_10_H_14_)_2_], contains one half of the centrosymmetric mol­ecule. Each Ru center is coordinated by tetra­methyl­benzene ring in a η^6^-coordination mode, and one terminal and two bridging bromine atoms. The aromatic rings and the Ru_2_Br_2_ four-membered ring form a dihedral angle of 55.99 (8)°. In the crystal structure, weak inter­molecular C—H⋯Br inter­actions link mol­ecules into chains propagated in [001].

## Related literature

For our work on the synthesis and catalytic applications of ruthenium–arene complexes, see: Cerón-Camacho *et al.* (2006 ▶); Díaz Camacho *et al.* (2008 ▶). For related structures, see: González-Torres *et al.* (2009 ▶) and references therein. For details of the synthesis, see: Bennett *et al.* (1982 ▶).
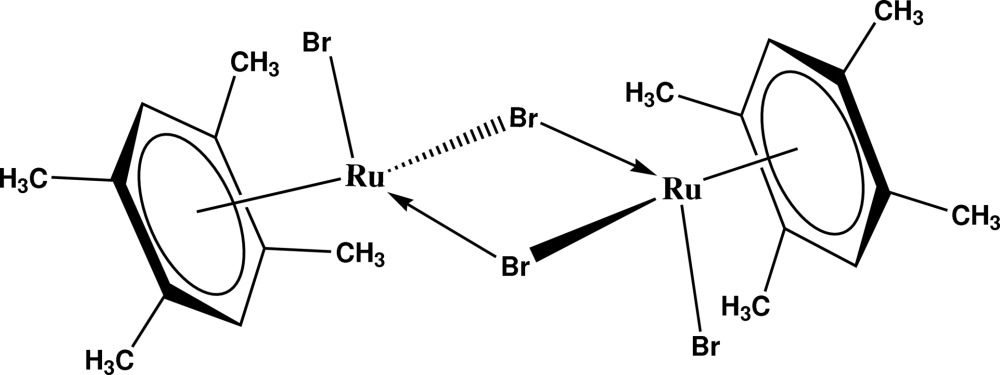


## Experimental

### 

#### Crystal data

[Ru_2_Br_4_(C_10_H_14_)_2_]
*M* *_r_* = 790.20
Triclinic, 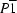

*a* = 7.8866 (13) Å
*b* = 8.2873 (14) Å
*c* = 9.8627 (17) Å
α = 88.335 (2)°
β = 74.508 (2)°
γ = 69.648 (2)°
*V* = 580.96 (17) Å^3^
*Z* = 1
Mo *K*α radiation
μ = 8.18 mm^−1^
*T* = 298 K
0.35 × 0.15 × 0.12 mm

#### Data collection

Bruker SMART CCD area-detector diffractometer
Absorption correction: face indexed-numerical (*SHELXTL*>; Sheldrick, 2008 ▶) *T*_min_ = 0.139, *T*_max_ = 0.478
4786 measured reflections
2108 independent reflections
1878 reflections with *I* > 2σ(*I*)
*R*_int_ = 0.063

#### Refinement

*R*[*F* ^2^ > 2σ(*F* ^2^)] = 0.024
*wR*(*F* ^2^) = 0.060
*S* = 1.00
2108 reflections
122 parameters
H-atom parameters constrained
Δρ_max_ = 0.52 e Å^−3^
Δρ_min_ = −0.65 e Å^−3^

### 

Data collection: *SMART* (Bruker, 1999 ▶); cell refinement: *SAINT* (Bruker, 1999 ▶); data reduction: *SAINT*; program(s) used to solve structure: *SHELXTL* (Sheldrick, 2008 ▶); program(s) used to refine structure: *SHELXTL*; molecular graphics: *SHELXTL*; software used to prepare material for publication: *SHELXTL*.

## Supplementary Material

Crystal structure: contains datablocks I, global. DOI: 10.1107/S1600536809049642/cv2636sup1.cif

Structure factors: contains datablocks I. DOI: 10.1107/S1600536809049642/cv2636Isup2.hkl

Additional supplementary materials:  crystallographic information; 3D view; checkCIF report

## Figures and Tables

**Table 1:** Hydrogen-bond geometry (Å, °)

*D*—H⋯*A*	*D*—H	H⋯*A*	*D*⋯*A*	*D*—H⋯*A*
C6—H6⋯Br2^i^	0.93	2.86	3.739 (4)	158

Symmetry code: (i) 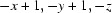.

**Fig. 1.**
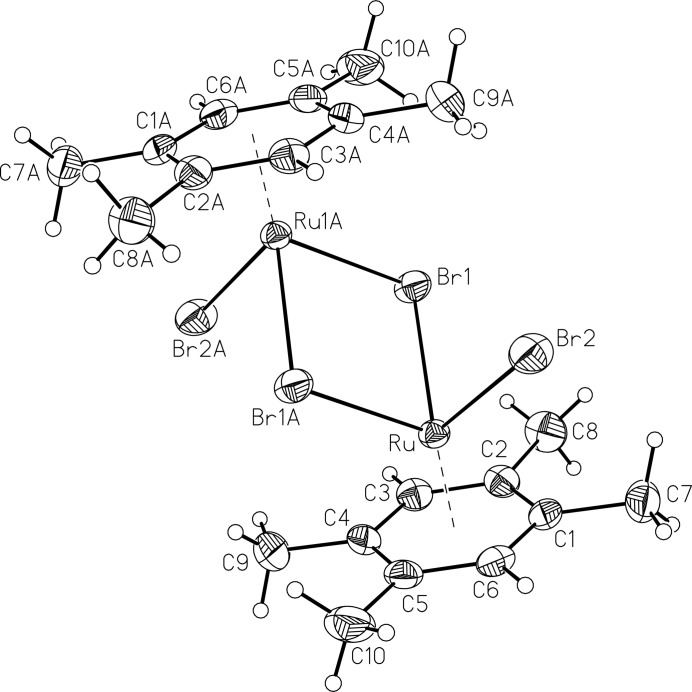
Molecular structure of (I) showing the atomic numbering and 40% probability displacement ellipsoids [symmetry code: (A) 1 - x, 1 - y, 1 - z].

## supplementary crystallographic information

### Comment

In continuation of our studies of ruthenium arene complexes (Díaz Camacho *et al.* 2008; Cerón-Camacho *et al.* 2006; González-Torres, *et al.* 2009), we present here the title compound (I).

The asymmetric unit of (I) consists of a half molecule, which is completed with a symmetry operation of 1 - *x*, 1 - *y*, 1 - *z* (Fig. 1). The bond lengths and angles in η^6^ - tetramethylbenzene fragment are in accordance with those observed in similar structures (González-Torres *et al.*, 2009 and references therein). Complex (I) exhibits a typical η^6^ - arene coordination of the tetramethylbenzene fragment to the ruthenium dinuclear structure bridged by two bromines and completing the coordination sphere two bromines one for each ruthenium atom arranged in a *trans* geometry. The η^6^ - arene fragment and the metal coordination center bridge (Br2—Ru—Br2 symmetry related) form a dihedral angle of 55.99 (8)°.

In the crystal structure, the molecules are linked by weak C—H···Br interaction (Table 1) into chains along direction [001].

### Experimental

The title compound was prepared according to the procedure reported by Bennett *et al.* (1982). Spectroscopic analysis agreed with that reported in the same reference.

### Refinement

C-bound H atoms were placed in geometrically idealized positions (C—H 0.93-0.96 Å), and refined as riding, with *U*_iso_(H) = 1.2-1.5 *U*_eq_ (C).

### Crystal data

**Table:**

[Ru_2_Br_4_(C_10_H_14_)_2_]	*Z* = 1
*M**_r_* = 790.20	*F*(000) = 376
Triclinic, *P*1	*D*_x_ = 2.259 Mg m^−^^3^
Hall symbol: -P 1	Mo *K*α radiation, λ = 0.71073 Å
*a* = 7.8866 (13) Å	Cell parameters from 3668 reflections
*b* = 8.2873 (14) Å	θ = 2.6–25.4°
*c* = 9.8627 (17) Å	µ = 8.18 mm^−^^1^
α = 88.335 (2)°	*T* = 298 K
β = 74.508 (2)°	Prism, red
γ = 69.648 (2)°	0.35 × 0.15 × 0.12 mm
*V* = 580.96 (17) Å^3^	

### Data collection

**Table:**

Bruker SMART CCD area-detector diffractometer	2108 independent reflections
Radiation source: fine-focus sealed tube	1878 reflections with *I* > 2σ(*I*)
graphite	*R*_int_ = 0.063
Detector resolution: 0.83 pixels mm^-1^	θ_max_ = 25.4°, θ_min_ = 2.2°
ω scans	*h* = −9→9
Absorption correction: face indexed-numerical (*SHELXTL*; Sheldrick, 2008)	*k* = −9→9
*T*_min_ = 0.139, *T*_max_ = 0.478	*l* = −11→11
4786 measured reflections	

### Refinement

**Table:**

Refinement on *F*^2^	Primary atom site location: structure-invariant direct methods
Least-squares matrix: full	Secondary atom site location: difference Fourier map
*R*[*F*^2^ > 2σ(*F*^2^)] = 0.024	Hydrogen site location: inferred from neighbouring sites
*wR*(*F*^2^) = 0.060	H-atom parameters constrained
*S* = 1.00	*w* = 1/[σ^2^(*F*_o_^2^) + (0.0227*P*)^2^] where *P* = (*F*_o_^2^ + 2*F*_c_^2^)/3
2108 reflections	(Δ/σ)_max_ = 0.001
122 parameters	Δρ_max_ = 0.52 e Å^−^^3^
0 restraints	Δρ_min_ = −0.65 e Å^−^^3^

### Special details

**Table:**

Geometry. All e.s.d.'s (except the e.s.d. in the dihedral angle between two l.s. planes) are estimated using the full covariance matrix. The cell e.s.d.'s are taken into account individually in the estimation of e.s.d.'s in distances, angles and torsion angles; correlations between e.s.d.'s in cell parameters are only used when they are defined by crystal symmetry. An approximate (isotropic) treatment of cell e.s.d.'s is used for estimating e.s.d.'s involving l.s. planes.
Refinement. Refinement of *F*^2^ against ALL reflections. The weighted *R*-factor *wR* and goodness of fit *S* are based on *F*^2^, conventional *R*-factors *R* are based on *F*, with *F* set to zero for negative *F*^2^. The threshold expression of *F*^2^ > σ(*F*^2^) is used only for calculating *R*-factors(gt) *etc*. and is not relevant to the choice of reflections for refinement. *R*-factors based on *F*^2^ are statistically about twice as large as those based on *F*, and *R*- factors based on ALL data will be even larger.

### Fractional atomic coordinates and isotropic or equivalent isotropic displacement parameters (Å^2^)

**Table:**

	*x*	*y*	*z*	*U*_iso_*/*U*_eq_	
Ru	0.63750 (4)	0.49125 (3)	0.30491 (3)	0.02810 (10)	
Br1	0.64758 (5)	0.29795 (4)	0.51434 (4)	0.04089 (12)	
Br2	0.41130 (6)	0.36687 (6)	0.24801 (4)	0.05137 (14)	
C1	0.8371 (5)	0.3925 (4)	0.0980 (4)	0.0367 (8)	
C2	0.9352 (5)	0.3568 (4)	0.2017 (4)	0.0379 (8)	
C3	0.9134 (5)	0.4943 (4)	0.2952 (4)	0.0377 (8)	
H3	0.9789	0.4699	0.3634	0.045*	
C4	0.7972 (5)	0.6654 (4)	0.2891 (4)	0.0407 (9)	
C5	0.6944 (5)	0.7014 (4)	0.1860 (4)	0.0396 (9)	
C6	0.7172 (5)	0.5650 (5)	0.0931 (4)	0.0389 (8)	
H6	0.6507	0.5888	0.0255	0.047*	
C7	0.8478 (6)	0.2543 (5)	−0.0040 (4)	0.0546 (11)	
H7A	0.7734	0.3062	−0.0672	0.082*	
H7B	0.8006	0.1717	0.0475	0.082*	
H7C	0.9762	0.1974	−0.0572	0.082*	
C8	1.0565 (6)	0.1754 (5)	0.2196 (5)	0.0557 (11)	
H8A	1.1764	0.1450	0.1505	0.084*	
H8B	0.9959	0.0967	0.2073	0.084*	
H8C	1.0744	0.1692	0.3124	0.084*	
C9	0.7843 (7)	0.8028 (5)	0.3909 (5)	0.0620 (12)	
H9A	0.8514	0.7506	0.4584	0.093*	
H9B	0.6547	0.8639	0.4392	0.093*	
H9C	0.8385	0.8818	0.3405	0.093*	
C10	0.5588 (7)	0.8778 (5)	0.1774 (5)	0.0629 (13)	
H10A	0.4830	0.8707	0.1176	0.094*	
H10B	0.6271	0.9520	0.1389	0.094*	
H10C	0.4792	0.9236	0.2700	0.094*	

### Atomic displacement parameters (Å^2^)

**Table:**

	*U*^11^	*U*^22^	*U*^33^	*U*^12^	*U*^13^	*U*^23^
Ru	0.02570 (16)	0.02940 (16)	0.02784 (16)	−0.01036 (12)	−0.00439 (11)	0.00292 (10)
Br1	0.0380 (2)	0.0341 (2)	0.0370 (2)	−0.00366 (16)	−0.00100 (16)	0.00683 (15)
Br2	0.0483 (3)	0.0736 (3)	0.0462 (2)	−0.0382 (2)	−0.0134 (2)	0.00497 (19)
C1	0.0333 (19)	0.0419 (19)	0.0313 (18)	−0.0173 (16)	0.0031 (15)	−0.0009 (15)
C2	0.0252 (17)	0.0367 (19)	0.045 (2)	−0.0104 (15)	0.0003 (15)	0.0011 (15)
C3	0.0300 (19)	0.045 (2)	0.042 (2)	−0.0177 (16)	−0.0095 (16)	0.0051 (16)
C4	0.045 (2)	0.0378 (19)	0.040 (2)	−0.0241 (17)	−0.0011 (17)	0.0014 (15)
C5	0.039 (2)	0.0362 (18)	0.040 (2)	−0.0169 (17)	−0.0010 (16)	0.0090 (15)
C6	0.041 (2)	0.049 (2)	0.0294 (17)	−0.0213 (18)	−0.0081 (16)	0.0099 (15)
C7	0.056 (3)	0.060 (3)	0.047 (2)	−0.029 (2)	0.001 (2)	−0.013 (2)
C8	0.038 (2)	0.046 (2)	0.069 (3)	−0.0035 (19)	−0.007 (2)	0.002 (2)
C9	0.084 (3)	0.057 (2)	0.056 (3)	−0.045 (3)	−0.008 (2)	−0.006 (2)
C10	0.066 (3)	0.043 (2)	0.065 (3)	−0.013 (2)	−0.006 (2)	0.021 (2)

### Geometric parameters (Å, °)

**Table:**

Ru—C6	2.150 (3)	C4—C5	1.427 (5)
Ru—C3	2.161 (4)	C4—C9	1.498 (5)
Ru—C5	2.180 (4)	C5—C6	1.408 (5)
Ru—C2	2.182 (3)	C5—C10	1.497 (5)
Ru—C1	2.191 (3)	C6—H6	0.9300
Ru—C4	2.200 (4)	C7—H7A	0.9600
Ru—Br2	2.5313 (6)	C7—H7B	0.9600
Ru—Br1^i^	2.5676 (5)	C7—H7C	0.9600
Ru—Br1	2.5780 (5)	C8—H8A	0.9600
Br1—Ru^i^	2.5676 (5)	C8—H8B	0.9600
C1—C2	1.407 (5)	C8—H8C	0.9600
C1—C6	1.421 (5)	C9—H9A	0.9600
C1—C7	1.511 (5)	C9—H9B	0.9600
C2—C3	1.421 (5)	C9—H9C	0.9600
C2—C8	1.510 (5)	C10—H10A	0.9600
C3—C4	1.406 (5)	C10—H10B	0.9600
C3—H3	0.9300	C10—H10C	0.9600
			
C6—Ru—C3	79.89 (15)	C4—C3—C2	122.8 (3)
C6—Ru—C5	37.93 (13)	C4—C3—Ru	72.7 (2)
C3—Ru—C5	68.14 (14)	C2—C3—Ru	71.7 (2)
C6—Ru—C2	68.16 (14)	C4—C3—H3	118.6
C3—Ru—C2	38.18 (13)	C2—C3—H3	118.6
C5—Ru—C2	81.73 (14)	Ru—C3—H3	129.7
C6—Ru—C1	38.21 (14)	C3—C4—C5	118.3 (3)
C3—Ru—C1	68.06 (13)	C3—C4—C9	119.3 (4)
C5—Ru—C1	69.31 (13)	C5—C4—C9	122.4 (3)
C2—Ru—C1	37.53 (14)	C3—C4—Ru	69.7 (2)
C6—Ru—C4	68.13 (14)	C5—C4—Ru	70.2 (2)
C3—Ru—C4	37.60 (13)	C9—C4—Ru	131.5 (3)
C5—Ru—C4	38.01 (14)	C6—C5—C4	118.6 (3)
C2—Ru—C4	69.00 (13)	C6—C5—C10	119.3 (4)
C1—Ru—C4	81.45 (13)	C4—C5—C10	122.0 (4)
C6—Ru—Br2	92.41 (11)	C6—C5—Ru	69.9 (2)
C3—Ru—Br2	154.25 (9)	C4—C5—Ru	71.7 (2)
C5—Ru—Br2	119.06 (11)	C10—C5—Ru	128.2 (3)
C2—Ru—Br2	116.19 (10)	C5—C6—C1	122.9 (3)
C1—Ru—Br2	90.67 (10)	C5—C6—Ru	72.2 (2)
C4—Ru—Br2	157.03 (10)	C1—C6—Ru	72.4 (2)
C6—Ru—Br1^i^	119.11 (10)	C5—C6—H6	118.5
C3—Ru—Br1^i^	118.25 (9)	C1—C6—H6	118.5
C5—Ru—Br1^i^	92.02 (9)	Ru—C6—H6	129.5
C2—Ru—Br1^i^	156.13 (10)	C1—C7—H7A	109.5
C1—Ru—Br1^i^	157.12 (10)	C1—C7—H7B	109.5
C4—Ru—Br1^i^	91.93 (9)	H7A—C7—H7B	109.5
Br2—Ru—Br1^i^	87.02 (2)	C1—C7—H7C	109.5
C6—Ru—Br1	158.03 (10)	H7A—C7—H7C	109.5
C3—Ru—Br1	90.73 (10)	H7B—C7—H7C	109.5
C5—Ru—Br1	152.74 (10)	C2—C8—H8A	109.5
C2—Ru—Br1	92.20 (10)	C2—C8—H8B	109.5
C1—Ru—Br1	119.82 (10)	H8A—C8—H8B	109.5
C4—Ru—Br1	115.15 (10)	C2—C8—H8C	109.5
Br2—Ru—Br1	87.508 (18)	H8A—C8—H8C	109.5
Br1^i^—Ru—Br1	82.838 (17)	H8B—C8—H8C	109.5
Ru^i^—Br1—Ru	97.162 (17)	C4—C9—H9A	109.5
C2—C1—C6	118.3 (3)	C4—C9—H9B	109.5
C2—C1—C7	122.8 (3)	H9A—C9—H9B	109.5
C6—C1—C7	118.8 (3)	C4—C9—H9C	109.5
C2—C1—Ru	70.89 (19)	H9A—C9—H9C	109.5
C6—C1—Ru	69.36 (18)	H9B—C9—H9C	109.5
C7—C1—Ru	129.3 (3)	C5—C10—H10A	109.5
C1—C2—C3	119.0 (3)	C5—C10—H10B	109.5
C1—C2—C8	121.6 (3)	H10A—C10—H10B	109.5
C3—C2—C8	119.3 (3)	C5—C10—H10C	109.5
C1—C2—Ru	71.58 (19)	H10A—C10—H10C	109.5
C3—C2—Ru	70.13 (19)	H10B—C10—H10C	109.5
C8—C2—Ru	128.1 (3)		
			
C6—Ru—Br1—Ru^i^	−177.6 (3)	Ru—C3—C4—C5	−52.5 (3)
C3—Ru—Br1—Ru^i^	118.39 (9)	C2—C3—C4—C9	−179.3 (4)
C5—Ru—Br1—Ru^i^	80.4 (2)	Ru—C3—C4—C9	127.1 (4)
C2—Ru—Br1—Ru^i^	156.56 (10)	C2—C3—C4—Ru	53.6 (3)
C1—Ru—Br1—Ru^i^	−176.65 (12)	C6—Ru—C4—C3	−102.2 (2)
C4—Ru—Br1—Ru^i^	88.77 (10)	C5—Ru—C4—C3	−132.1 (3)
Br2—Ru—Br1—Ru^i^	−87.31 (2)	C2—Ru—C4—C3	−28.2 (2)
Br1^i^—Ru—Br1—Ru^i^	0.0	C1—Ru—C4—C3	−64.9 (2)
C6—Ru—C1—C2	131.8 (3)	Br2—Ru—C4—C3	−136.0 (2)
C3—Ru—C1—C2	29.9 (2)	Br1^i^—Ru—C4—C3	137.1 (2)
C5—Ru—C1—C2	103.8 (2)	Br1—Ru—C4—C3	54.1 (2)
C4—Ru—C1—C2	66.5 (2)	C6—Ru—C4—C5	29.92 (19)
Br2—Ru—C1—C2	−135.2 (2)	C3—Ru—C4—C5	132.1 (3)
Br1^i^—Ru—C1—C2	140.9 (2)	C2—Ru—C4—C5	103.9 (2)
Br1—Ru—C1—C2	−47.7 (2)	C1—Ru—C4—C5	67.2 (2)
C3—Ru—C1—C6	−101.9 (2)	Br2—Ru—C4—C5	−3.9 (4)
C5—Ru—C1—C6	−27.9 (2)	Br1^i^—Ru—C4—C5	−90.81 (19)
C2—Ru—C1—C6	−131.8 (3)	Br1—Ru—C4—C5	−173.80 (16)
C4—Ru—C1—C6	−65.3 (2)	C6—Ru—C4—C9	146.2 (4)
Br2—Ru—C1—C6	93.0 (2)	C3—Ru—C4—C9	−111.6 (5)
Br1^i^—Ru—C1—C6	9.1 (4)	C5—Ru—C4—C9	116.3 (5)
Br1—Ru—C1—C6	−179.45 (18)	C2—Ru—C4—C9	−139.8 (4)
C6—Ru—C1—C7	−111.0 (4)	C1—Ru—C4—C9	−176.5 (4)
C3—Ru—C1—C7	147.2 (4)	Br2—Ru—C4—C9	112.4 (4)
C5—Ru—C1—C7	−138.9 (4)	Br1^i^—Ru—C4—C9	25.5 (4)
C2—Ru—C1—C7	117.2 (4)	Br1—Ru—C4—C9	−57.5 (4)
C4—Ru—C1—C7	−176.3 (4)	C3—C4—C5—C6	−1.4 (5)
Br2—Ru—C1—C7	−17.9 (3)	C9—C4—C5—C6	179.0 (3)
Br1^i^—Ru—C1—C7	−101.9 (4)	Ru—C4—C5—C6	−53.7 (3)
Br1—Ru—C1—C7	69.6 (4)	C3—C4—C5—C10	176.4 (4)
C6—C1—C2—C3	−1.2 (5)	C9—C4—C5—C10	−3.2 (6)
C7—C1—C2—C3	−178.6 (3)	Ru—C4—C5—C10	124.1 (4)
Ru—C1—C2—C3	−53.6 (3)	C3—C4—C5—Ru	52.3 (3)
C6—C1—C2—C8	176.3 (4)	C9—C4—C5—Ru	−127.3 (4)
C7—C1—C2—C8	−1.1 (6)	C3—Ru—C5—C6	102.0 (2)
Ru—C1—C2—C8	123.9 (4)	C2—Ru—C5—C6	64.8 (2)
C6—C1—C2—Ru	52.4 (3)	C1—Ru—C5—C6	28.1 (2)
C7—C1—C2—Ru	−125.0 (4)	C4—Ru—C5—C6	131.1 (3)
C6—Ru—C2—C1	−29.8 (2)	Br2—Ru—C5—C6	−50.6 (2)
C3—Ru—C2—C1	−131.6 (3)	Br1^i^—Ru—C5—C6	−138.3 (2)
C5—Ru—C2—C1	−66.6 (2)	Br1—Ru—C5—C6	143.5 (2)
C4—Ru—C2—C1	−103.8 (2)	C6—Ru—C5—C4	−131.1 (3)
Br2—Ru—C2—C1	51.8 (2)	C3—Ru—C5—C4	−29.2 (2)
Br1^i^—Ru—C2—C1	−142.7 (2)	C2—Ru—C5—C4	−66.3 (2)
Br1—Ru—C2—C1	140.1 (2)	C1—Ru—C5—C4	−103.0 (2)
C6—Ru—C2—C3	101.8 (2)	Br2—Ru—C5—C4	178.27 (16)
C5—Ru—C2—C3	64.9 (2)	Br1^i^—Ru—C5—C4	90.55 (19)
C1—Ru—C2—C3	131.6 (3)	Br1—Ru—C5—C4	12.3 (3)
C4—Ru—C2—C3	27.8 (2)	C6—Ru—C5—C10	112.1 (5)
Br2—Ru—C2—C3	−176.69 (17)	C3—Ru—C5—C10	−146.0 (4)
Br1^i^—Ru—C2—C3	−11.2 (4)	C2—Ru—C5—C10	176.9 (4)
Br1—Ru—C2—C3	−88.4 (2)	C1—Ru—C5—C10	140.2 (4)
C6—Ru—C2—C8	−146.0 (4)	C4—Ru—C5—C10	−116.8 (4)
C3—Ru—C2—C8	112.3 (4)	Br2—Ru—C5—C10	61.5 (4)
C5—Ru—C2—C8	177.2 (4)	Br1^i^—Ru—C5—C10	−26.2 (4)
C1—Ru—C2—C8	−116.2 (4)	Br1—Ru—C5—C10	−104.5 (4)
C4—Ru—C2—C8	140.0 (4)	C4—C5—C6—C1	0.4 (5)
Br2—Ru—C2—C8	−64.4 (4)	C10—C5—C6—C1	−177.4 (4)
Br1^i^—Ru—C2—C8	101.1 (4)	Ru—C5—C6—C1	−54.1 (3)
Br1—Ru—C2—C8	23.9 (3)	C4—C5—C6—Ru	54.6 (3)
C1—C2—C3—C4	0.2 (5)	C10—C5—C6—Ru	−123.3 (4)
C8—C2—C3—C4	−177.4 (4)	C2—C1—C6—C5	0.9 (5)
Ru—C2—C3—C4	−54.1 (3)	C7—C1—C6—C5	178.4 (3)
C1—C2—C3—Ru	54.3 (3)	Ru—C1—C6—C5	54.0 (3)
C8—C2—C3—Ru	−123.3 (3)	C2—C1—C6—Ru	−53.1 (3)
C6—Ru—C3—C4	67.1 (2)	C7—C1—C6—Ru	124.4 (3)
C5—Ru—C3—C4	29.5 (2)	C3—Ru—C6—C5	−67.3 (2)
C2—Ru—C3—C4	134.5 (3)	C2—Ru—C6—C5	−105.2 (2)
C1—Ru—C3—C4	105.1 (2)	C1—Ru—C6—C5	−134.5 (3)
Br2—Ru—C3—C4	141.4 (2)	C4—Ru—C6—C5	−30.0 (2)
Br1^i^—Ru—C3—C4	−50.6 (2)	Br2—Ru—C6—C5	137.5 (2)
Br1—Ru—C3—C4	−132.8 (2)	Br1^i^—Ru—C6—C5	49.5 (2)
C6—Ru—C3—C2	−67.4 (2)	Br1—Ru—C6—C5	−133.2 (2)
C5—Ru—C3—C2	−105.0 (2)	C3—Ru—C6—C1	67.2 (2)
C1—Ru—C3—C2	−29.4 (2)	C5—Ru—C6—C1	134.5 (3)
C4—Ru—C3—C2	−134.5 (3)	C2—Ru—C6—C1	29.3 (2)
Br2—Ru—C3—C2	6.9 (4)	C4—Ru—C6—C1	104.5 (2)
Br1^i^—Ru—C3—C2	174.90 (17)	Br2—Ru—C6—C1	−88.0 (2)
Br1—Ru—C3—C2	92.6 (2)	Br1^i^—Ru—C6—C1	−175.95 (18)
C2—C3—C4—C5	1.1 (5)	Br1—Ru—C6—C1	1.3 (4)

Symmetry codes: (i) −*x*+1, −*y*+1, −*z*+1.

### Hydrogen-bond geometry (Å, °)

**Table:**

*D*—H···*A*	*D*—H	H···*A*	*D*···*A*	*D*—H···*A*
C6—H6···Br2^ii^	0.93	2.86	3.739 (4)	158

Symmetry codes: (ii) −*x*+1, −*y*+1, −*z*.
